# 

**Published:** 1886-07

**Authors:** 


					﻿-
1
JULY, 1836
FOUR ADDITIONAL PAGES.
OLD SERIES
VoL XXV
& 1855
NEW SERIES .
Vol. III.—No. 5- \J
_	1039

tbl.x.11 ~Wi“	rx-jji. cYif w
L?ye4by* W.F.WESTMORElTAmWF
M?A h.v.m.miller.m.dj.l.dJ^
JAMES A.GRAY, MU/M
*> G —$ J
Published By james rharrison&co
p ____________Atlanta .ga. < JaM
SUBSCRIPTION: S2.DO A YEAR.
				

